# Modified Low-Temperature Extraction Method for Isolation of *Bletilla striata* Polysaccharide as Antioxidant for the Prevention of Alzheimer’s Disease

**DOI:** 10.3390/ijms222312760

**Published:** 2021-11-25

**Authors:** Yi-Wen Lin, Chih-Hsiang Fang, Ya-Jyun Liang, Hong-Hsiang Liao, Feng-Huei Lin

**Affiliations:** 1 Institute of Biomedical Engineering, National Taiwan University, Taipei 10051, Taiwan; zhew520@gmail.com (Y.-W.L.); kittenor210@gmail.com (Y.-J.L.); hung.ming.is.the.best@gmail.com (H.-H.L.); 2 Trauma and Emergency Center, China Medical University Hospital, Taichung City 404332, Taiwan; danny07291991@hotmail.com; 3 Institute of Biomedical Engineering and Nanomedicine Research, National Health Research Institutes, No. 35, Keyan Road, Miaoli County 35053, Taiwan

**Keywords:** Alzheimer’s disease, *Bletilla striata*, amyloid-β, oxidative stress, anti-inflammation, aluminum chloride

## Abstract

Amyloid-β (Aβ) peptides play a key role in Alzheimer’s disease (AD), the most common type of dementia. In this study, a polysaccharide from *Bletilla striata* (BSP), with strong antioxidant and anti-inflammatory properties, was extracted using a low-temperature method and tested for its efficacy against AD, in vitro using N2a and BV-2 cells, and in vivo using an AD rat model. The characterization of the extracted BSP for its molecular structure and functional groups demonstrated the effectiveness of the modified method for retaining its bioactivity. In vitro, BSP reduced by 20% reactive oxygen species (ROS) levels in N2a cells (*p* = 0.0082) and the expression levels of inflammation-related genes by 3-fold TNF-α (*p* = 0.0048), 4-fold IL-6 (*p* = 0.0019), and 2.5-fold IL-10 (*p* = 0.0212) in BV-2 cells treated with Aβ fibrils. In vivo, BSP recovered learning memory, ameliorated morphological damage in the hippocampus and cortex, and reduced the expression of the β-secretase protein in AlCl_3_-induced AD rats. Collectively, these findings demonstrated the efficacy of BSP for preventing and alleviating the effects of AD.

## 1. Introduction

Alzheimer’s disease (AD) is the most common type of dementia, characterized by progressive cognitive and memory loss accompanied by personality changes [1,2]. Given the expected increase in the aged population globally, the prevalence of dementia has been projected to increase to 131 million by 2050 [2]. This impending rise in societal aging could impose a costly social impact in the coming years.

Several studies have demonstrated that the initial changes in brain physiology associated with AD begin years before perceptible symptoms of dementia are observed. In the initial stages, the brain compensates for the changes enabling affected individuals to function normally. However, with the progress of neuronal damage, the brain fails to compensatefurther, and individuals show subtle cognitive decline [3,4]. AD is generally diagnosed by positron emission tomography (PET) and cerebrospinal fluid (CSF) tests [5] that reveal the elevated amyloid-β (Aβ) depositions, which are believed to be related to pathological changes in preclinical AD.

Several biomarkers detecting specific proteins in blood or body fluids, which could be correlated to Aβ deposition, pathologic tau, and neurodegeneration, are also used to uncover the pathological process of AD [6]. These include p53-misfolding conformation, also called Unfolded p53 (U-p532D3A8+), recognized by the antibody 2D3A8. According to Abate, G. et al., p53 misfolded variants may represent the characteristics of early AD pathological events, including Aß accumulation, and redox imbalance, and immune activation, which ultimately lead to oxidative stress and chronic inflammation, respectively. Thus, U-p532D3A8+ plasma levels are a promising blood-based candidate biomarker for AD [7].

In addition, there may be a link between the gut microbiota and neurodegenerative diseases, such as Parkinson’s disease and amyotrophic lateral sclerosis [8,9]. The microbiota–gut–brain axis has become the focus of biomedical research and a potential therapeutic target for central nervous system diseases [10,11]. In particular, the dysfunction of the microbiota-gut-brain axis is related to the pathogenesis of AD [12]. The gut microbiota is considered a forgotten regulator of host physiology and metabolism [13]. The influence of the microbiota extends to brain development and cognitive functions [14].

Aβ is derived from amyloid precursor protein (APP) by a two-step proteolysis reaction of two enzyme complexes, β-secretase and γ-secretase [15]. It has been reported that the accumulation and aggregation of Aβ leading to the formation of senile plaques in the central nervous system causes progressive cognitive memory loss. Furthermore, Aβ can also increase the levels of reactive oxidative species (ROS) that induces oxidative stress to the neurons and cause cytotoxicity. In addition, the senile plaques containing the Aβ peptide have also been shown to induce oxidative stress and chronic neuroinflammation leading to neurotoxicity, which further advances disease progression. 

Studies have shown that microglia that secrete neuroprotective growth factors and anti-inflammatory cytokines phagocytize Aβ and release enzymes responsible for Aβ degradation; they delay the progression of AD by contributing to the clearance of Aβ [16,17]. Therefore, it is predicted that finding a way to reduce the oxidative stress and chronic neuroinflammation might slow down the progression of AD or even stop the accumulation of Aβ. However, despite the availability of several reports and significant efforts by pharmaceutical industries to understand the mechanism, progression, and alleviation of AD, effective therapies to cure AD or inhibit the progression of AD symptoms significantly are unavailable [18]. Moreover, the drugs currently used to treat AD—donepezil, galantamine, and rivastigmine—mainly focus on anticholinesterase activity rather than ROS and neuroinflammation reduction.

*Bletilla striata* (Thunb.) Reichb. f. (Orchidaceae), a traditional Chinese medicine, has been widely used in East Asian countries for alimentary canal damage, ulcers, bleeding, bruises, and burns [19]. *B. striata* polysaccharide (BSP) is a water-soluble polysaccharide isolated from *B. striata.* The monosaccharides of BSP are composed of (1→2)-linked α-D-mannopyranose and (1→4)-linked β-D-glucopyranose residues in a ratio of 3:1 [20]. Studies have reported that BSP could scavenge the ROS and inhibit the activation of proinflammatory cytokines, including interleukin 6 (IL-6) and tumor necrosis factor α (TNF-α) in a dose–dependent manner [21]. Therefore, we hypothesized that BSP with strong anti-oxidant and anti-inflammatory properties could be effective in preventing AD. 

**Figure 1:**
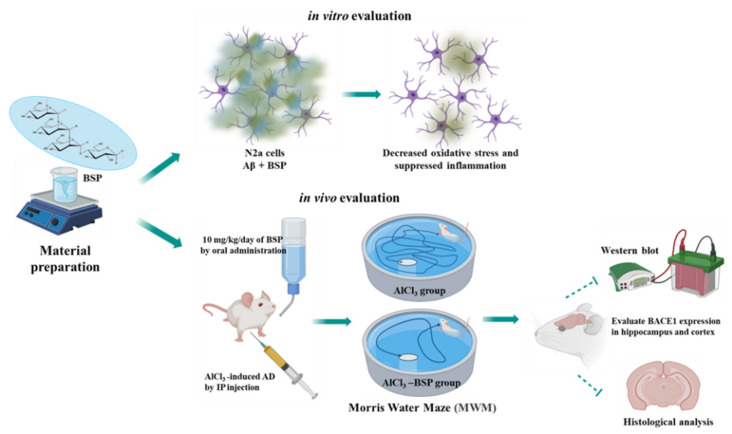
The scheme of the experimental design. This study used the N2a cell as an in-vitro model to investigate the decrease of amyloid-β plaque by BSP. In addition, the AD rat model was induced by intraperitoneal (IP) injection of AlCl_3_ three times a week, and the Morris water maze was used to assess working and spatial memory retention in the rats. Finally, the biochemical analysis was used to evaluate the effect of AD prevention by BSP.

To test this hypothesis, in this study, we investigated whether BSP could prevent the diffusion of ROS from Aβ in vitro and the possibility of recovery from the disease by memory improvement in the AlCl_3_-induced AD rat model in vivo (Figure 1). Furthermore, here, we employed a new BSP extraction method at low temperatures to ensure the retention of its bioactive properties. 

## 2. Results

### 2.1. Identification of Molecular Structure and Functional Group

**Figure 2:**
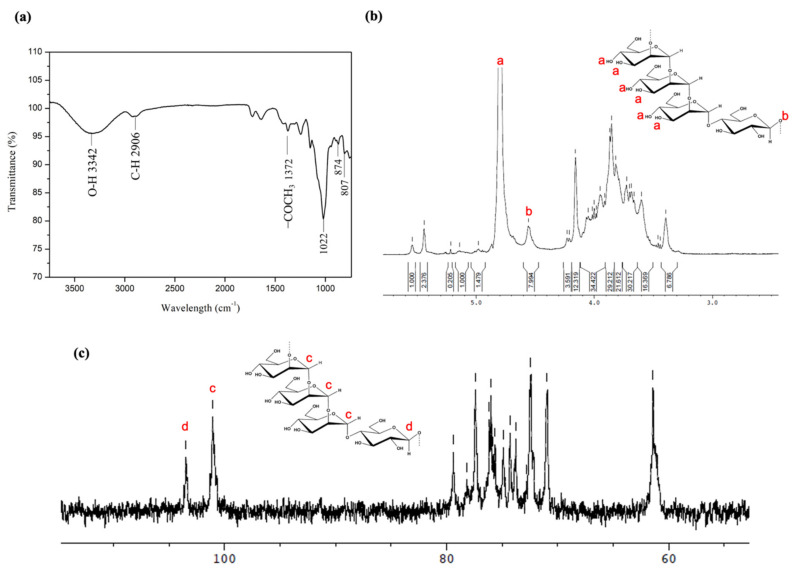
Characterization of extracted BSP. (**a**) FTIR spectrum; (**b**) ^1^H NMR spectrum; (**c**) ^13^C NMR spectrum. These were fully matched to the BSP extracted by the traditional method.

In the study, BSP was extracted from *B. striata* at low temperature combined with the use of vacuum system to retain the bioactivity of BSP and to reduce protein extraction. Figure 2a shows the FTIR pattern of the extracted BSP obtained. The absorption bands at 807 cm^−1^ and 874 cm^−1^ represented α-mannose residues, whereas the sharp absorption at 1022 cm^−1^ indicated a β-glucose residue. The absorption bands at 1372 cm^−1^, 2906 cm^−1^, and 3342 cm^−1^ corresponded to the C-O-C of glycosidic linkage, C-H of the 6-carbon ring, and the hydroxyl group (O−H), respectively. Furthermore, as shown in Figure 2b, the pattern of ^1^H NMR of the extracted BSP revealed a chemical shift at δ 4.80 ppm and δ 4.55 ppm, the characteristic peaks for α-mannopyranose and β-glucopyranose, respectively. The signals in the other chemical shifts in the range of 3.3–4.2 ppm corresponded to the non-anomeric ring protons. The 13C NMR pattern of extracted BSP represented the signals at δ 101.1 ppm and δ 103.5 ppm, attributed to α-mannopyranose and β-glucopyranose, respectively, whereas the chemical shifts found in the range of 70–80 ppm indicated the non-anomeric carbon rings (Figure 2c). The results of ^1^H NMR and ^13^C NMR are in agreement with the results of a previous study obtained by fingerprinting analysis [20], suggesting that the molecular structure and functional groups of BSP extracted by the new method proposed in this study were the same as for the BSP extracted by traditional method.

### 2.2. BSP Inhibit Aβ Fibril Formation

**Figure 3:**
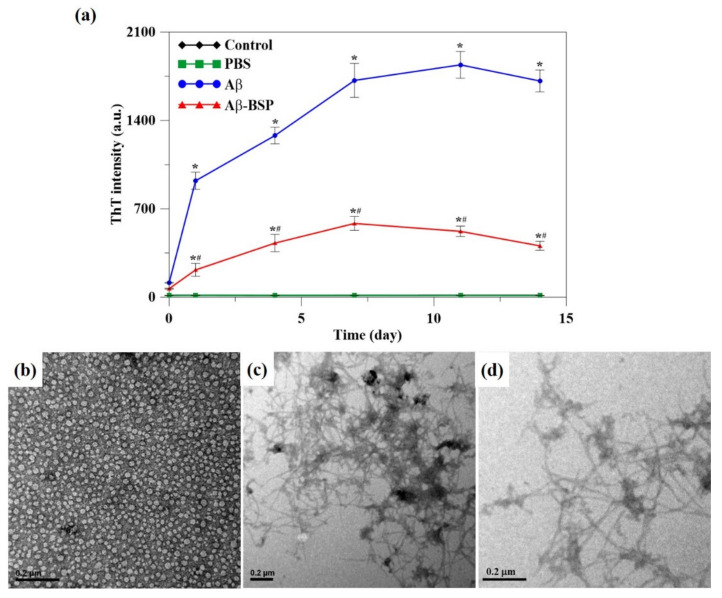
The aggregation of Aβ42 into Aβ fibrils. (**a**) The fluorescence intensity of ThT was obtained by incubation of Aβ42 (Aβ; blue line), Aβ42 mixed with BSP (Aβ-BSP; red line) in PBS at 37 °C for 14 days. The green line represents the fluorescence intensity of ThT in PBS only (PBS). (**b**) The TEM image of Aβ42 monomers without aggregation on day 0; (**c**) the Aβ42 aggregated into thick and compact Aβ fibrils on day 7; (**d**) BSP reduced aggregation of Aβ42 with reduced thickness and loose packing of Aβ fibrils on day 7. The scale bar is 0.2 μm. (*n* = 6, # *p* < 0.05 compared to control group, * *p* < 0.05 compared to Aβ group).

It is known that Aβ42 in PBS gradually aggregates into the Aβ fibril, which, upon binding to Thioflavin T (ThT), emits fluorescence. As shown in Figure 3a, neither the control nor the PBS alone showed any fluorescence, which increased markedly with time when ThT was incubated with Aβ42 and Aβ42 mixed with BSP (Aβ-BSP) in PBS at 37 °C as compared to that in PBS alone. However, the intensity of the emitted fluorescence was markedly lower with Aβ-BSP treatment than that obtained by Aβ42, indicating the inhibitory effects of BSP on Aβ fibril formation or Aβ42 aggregation.

In addition, TEM imaging data showed that in cells treated with Aβ42 (in PBS), Aβ42 did not aggregate on day 0 but formed thick Aβ fibrils on day 7 (Figure 3b,c). In contrast, in the Aβ-BSP-treated cells, the aggregation of Aβ42 was relatively loose, and the number of Aβ fibrils was less than that observed in the Aβ42-treated cells on day 7 (Figure 3d). These findings further support the findings obtained through ThT assays. In summary, it can be inferred that BSP effectively inhibits Aβ42 aggregation to block the formation of Aβ fibrils.

### 2.3. Effects of BSP on Aβ-Induced Cytotoxicity and Cell Viability

**Figure 4:**
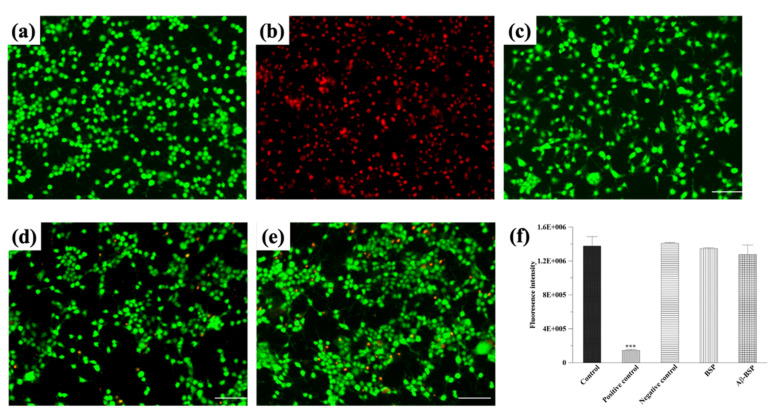
The LIVE/DEAD staining of N2a cells in (**a**) the control: cells cultured in medium only, (**b**) positive control: cells treated with zinc diethyldithiocarbamate, (**c**) negative control: cells treated with aluminum oxide, (**d**) BSP: cells treated with an extracted solution of BSP, and (**e**) Aβ-BSP: cells treated with Aβ42 and BSP. (**f**) Quantitative results of survival rate in N2a cells. *** *p* < 0.001 compared with control.

The protective effects of BSP against Aβ-induced cytotoxicity on N2a cells were assessed by LIVE/DEAD staining. As shown in Figure 4, the survivability of the N2a cells treated with BSP and Aβ42+BSP were not significantly different from that observed in untreated controls. However, the numbers of dead cells in BSP-only treated cells were lower than those treated with Aβ42-BSP, indicating the cytotoxic effects were induced by Aβ fibrils in N2a cells.

**Figure 5:**
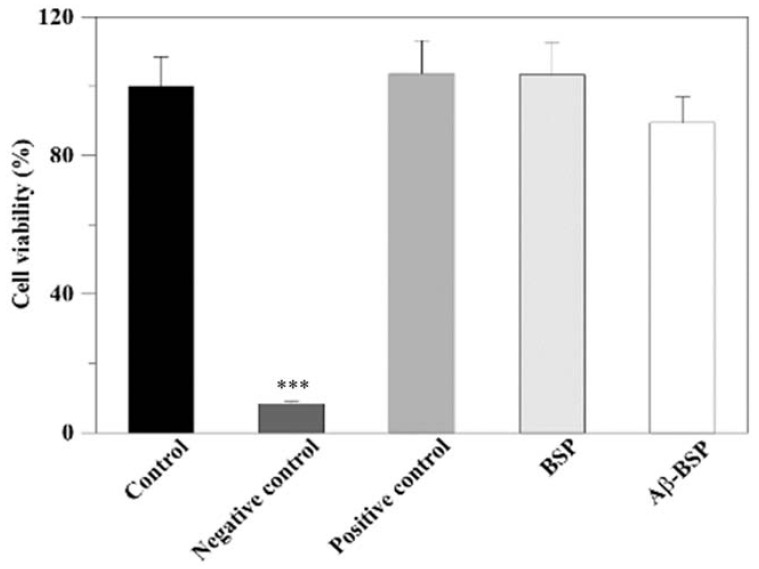
The effects of the extracted BSP on cell viability were estimated by the WST-1 assay. (*n* = 6, *** *p* < 0.001 compared with control). The abbreviations are as defined in Figure 4.

Furthermore, the effects of extracted BSP on the viability of N2a cells evaluated by WST-1 assay revealed that neither the extracted BSP alone nor that mixed with Aβ42 showed any toxicity to N2a cells compared to the control (Figure 5).

### 2.4. Antioxidant Activity of BSP

**Figure 6:**
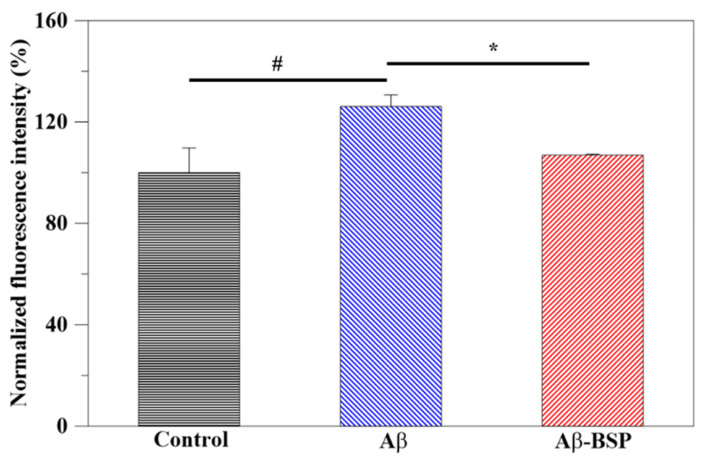
Antioxidant activity of extracted BSP. DCFDA was used to measure the intracellular ROS in N2a cells without any treatment (control), treated with Aβ42 (Aβ), and Aβ42 and BSP (Aβ42-BSP). (*n* = 6, # *p* < 0.05 compared to control group, * *p* < 0.05 compared to Aβ group).

The antioxidant ability of the extracted BSP was evaluated by DCFDA assay. As shown in Figure 6, the treatment of Aβ42 alone significantly increased the ROS level in N2a cells cultured for 1 day relative to the control that did not receive any treatment (*p* < 0.05), whereas that in Aβ42- and BSP-treated cells did not vary. However, the ROS level in Aβ-BSP treated cells was significantly reduced compared to those treated with Aβ42 alone, indicating the potential of BSP to ameliorate the Aβ-induced increase in ROS levels.

### 2.5. Anti-Inflammatory Activity of BSP

**Figure 7:**
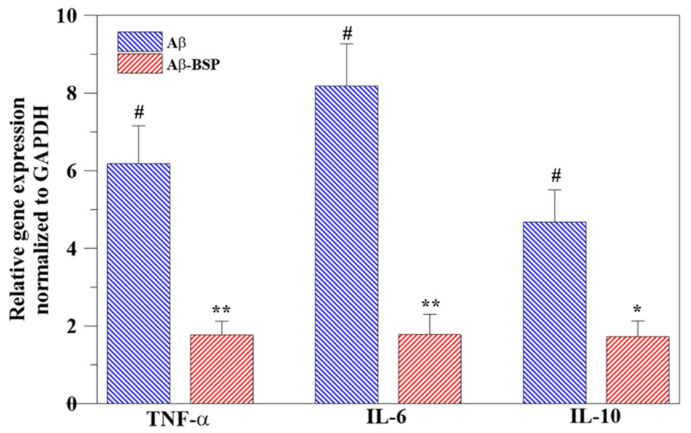
Anti-inflammatory effect of the extracted BSP on Aβ-induced inflammation in BV-2 cells estimated by the expression analysis of inflammation-related genes, TNF-α, IL-6, and IL-10. (*n* = 6, # *p* < 0.05 compared to that in the control group, * *p* < 0.05 compared to Aβ group, ** *p* < 0.01 compared to Aβ group).

As shown in Figure 7, expression of all three inflammatory genes—TNF-α, IL-6, and IL-10—was downregulated in the BV-2 cells in the Aβ-BSP group compared to those in the Aβ group, suggesting the strong anti-inflammatory properties of BSP (Figure 7).

### 2.6. Morris Water Maze Test

**Figure 8:**
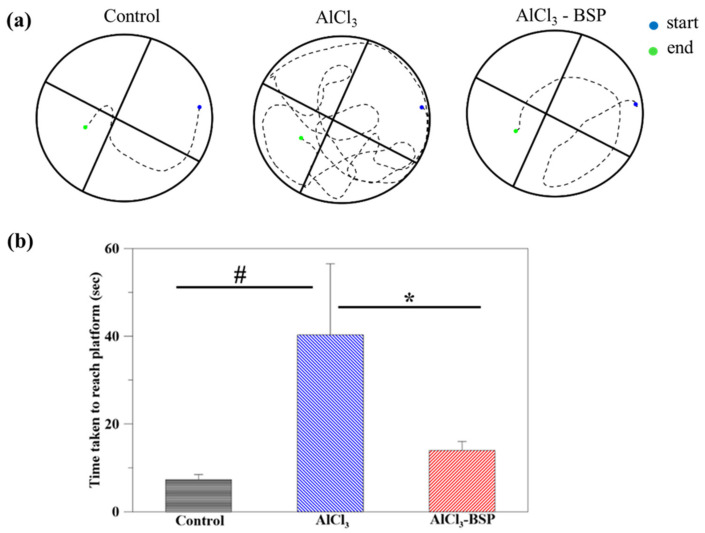
The MWM track of the normal rats (control), AlCl_3_-induced AD rats (AlCl_3_), and BSP-treated AD rats (AlCl_3_-BSP). (**a**) The starting point (blue) to endpoint (green) of all the tested rats was the same. (**b**) The time taken by the tested rats from starting point to reach the escape platform (*n* = 6 per group, # *p* < 0.05 compared to control group, * *p* < 0.05 compared to AlCl_3_ group).

As a next step, we evaluated the effects of BSP in vivo. First, we evaluated its effects on the working and spatial memory of rats. As shown in Figure 8a, a normal rat with a good memory reached the escape platform following a relatively straight track (Figure 8a), whereas an AlCl_3_-induced AD rat could not reach the escape platform even after following a complex track. However, the track followed by a BSP-treated AD rat was like that of a normal rat. Furthermore, the findings revealed that the time required by the BSP group did not differ significantly compared to the time required by the control group (Figure 8b). Collectively, these results indicate the potential of BSP to restore memory in AD rats.

### 2.7. BSP Treatment Downregulated the BACE1 Treatment

**Figure 9:**
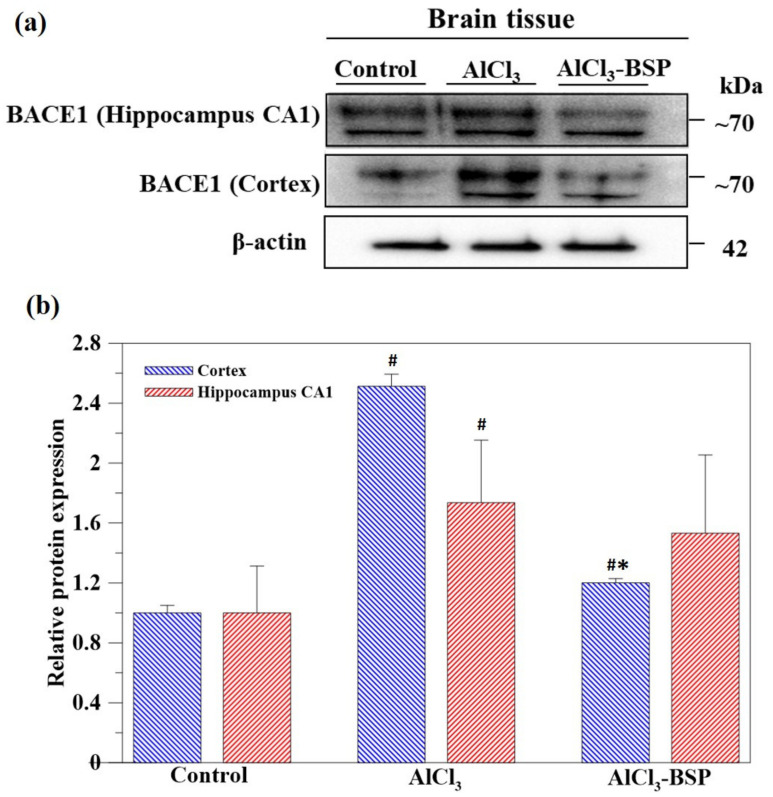
Bio-chemical analysis. (**a**) The western blot analysis of BACE1 in hippocampus CA1 and cortex. β-actin was used as the loading control. (**b**) The quantitative values of the BACE1 expression data obtained the ratio of BACE1 protein/actin protein band intensities normalized to 1 in the control group. (*n* = 6, # *p* < 0.05 compared to control group, * *p* < 0.05 compared to AlCl_3_ group).

To investigate the Aβ fibril formations in AlCl_3_-induced rats, the cortex and hippocampus were harvested from the brain tissue, and the expression of the β-secretase (BACE1) protein was analyzed by western blotting (Figure 9a). As shown in Figure 9, both in the cortex and hippocampus, BACE1 was highly expressed in AlCl_3_-induced rats, which was downregulated in BSP-treated AD rats. The measured values of the band intensities are shown in Figure 9b.

### 2.8. BSP Treatment Reduced Morphological Changes in the Neurons of the AD Rat Model

**Figure 10:**
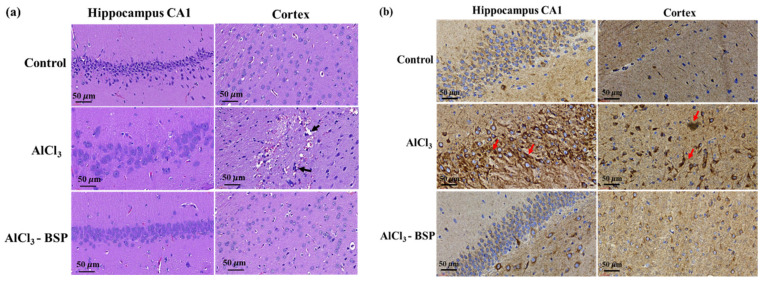
Histological staining. (**a**) Hematoxylin and eosin (H&E) staining of hippocampal CA1 and cortex regions of the tested rats. Brain sections from rats treated with AlCl_3_ show that pyramidal cells in the hippocampal CA1 region exhibit more severe morphological changes. (**b**) BACE1 reveals plaque-like staining in the brains of AD rats by immunohistochemistry. Brain sections from control rats stained with BACE1 show the normal BACE1 immunoreactivity pattern, whereas those from AlCl_3_-induced rats display plaque-like BACE1 immunoreactivity, which was reduced in BSP-treated AD rats. Arrowheads indicate plaque-like BACE1-immunopositive deposits. Scale bars: 50 μm.

H&E staining was used to assess the effects of BSP on morphological changes of the cells in the cortex and hippocampus of the AlCl_3_-induced AD rat. As shown in Figure 10a, the hippocampal CA1 and cerebral cortex regions in the control group showed normal morphology, whereas the cells from AlCl_3_-induced AD rats showed clear damage, such as cellular atrophy, shrinkage, necrosis, and pyknosis, indicated by heavy stained and dark nuclei (hyperchromatic cells). In addition, large cells due to neuronal swelling and vacuolation were also observed sporadically. In contrast, the damage was substantially decreased in AlCl_3_-induced AD rats treated with the extracted BSP, indicating the ameliorating effects of BSP against the damages induced by AlCl_3_ in AD rats. As shown in Figure 10b, the dark-brown amyloid plaques were observed in the hippocampus CA1 and cortex of AlCl_3_-induced rats by BACE1 antibody-positive stain (indicated by the red arrows). However, no plaques were detected in the control rats, whereas they were barely traced or observed in very light stains in AlCl_3_-induced AD rats treated with BSP.

## 3. Discussion

In this study, the BSP was successfully isolated and purified from the tubers of *B. striata* following a modified extraction method at low temperature that could effectively preserve BSP bioactivity and reduce protein loss. We obtained a new polysaccharide with a molecular weight of 2.35 × 10^5^ Da containing mannose, glucose, and galactose in a molar ratio of 9.4:2.6:1.0. The results indicated that the backbone of BSP consisted of (1→4)-linked mannosyl residues and (1→4)-linked glucosyl residues in a molar ratio of 2:1. A monosaccharide composition analysis revealed multiple repetitive sequences. The molecular structure and functional groups of the extracted BSP characterized by NMR and FTIR are fully in agreement with that obtained by the traditional method [20]. Furthermore, the extracted BSP demonstrated good survivability and no harm to the N2a cells in vitro. It also alleviated the damage to the hippocampus and cortex and recovered the neurodegeneration in AlCl_3_-induced AD rats in vivo.

Aluminum treatment increased Aβ(1–42) levels in the brain by up-regulating BACE1 expression [22]. In addition, aluminum treatment inhibited the expression of LRP1 and NEP (Aβ clearance related protein) [23]. The present study demonstrated the aggregation of Aβ42 into Aβ fibrils in Aβ42 in BSP-treated cells, which could be because of the Aβ42 binding to the ThT, which restricts the rotation of the benzylamine and benzothiazole rings of ThT, consequently emitting fluorescence. Furthermore, it has been shown that the deposition of Aβ fibrils as plaques are enriched with the β-sheet structure, which blocks neurotransmitter transportation, consequently leading to AD [24]. The findings showed that the formation of Aβ fibrils was reduced by treating the cells with BSP, which could be attributed to the inhibition of the aggregation of Aβ42 due to the stereo-obstacles. Meanwhile, our results also indicated that BSP could down-regulate BACE1 expression. Therefore, BSP might be a potential supplement for AD prevention.

It has been reported that microglia surrounding the Aβ fibrils form a protective barrier around them, limit further recruitment of Aβ, and release enzymes responsible for Aβ fibril degradation that delay the progression of AD [25]. Furthermore, it has been established that Aβ plays a significant role in inducing and regulating microglial ROS production. Although the breakdown by microglial proteases could enhance the clearance of Aβ deposits, the clearance rate is relatively slow for diffusing the stress from deposited Aβ fibrils fully, consequently, over years, this leads to accumulation of Aβ fibrils resulting in induction of ROS and chronic inflammation [26,27]. In addition, the production of ROS by microglial cells and subsequent oxidative stress are strongly implicated in the pathogenesis of AD. The oxidative stress to the surrounding cells subsequently induces chronic inflammation, which might cause further damage to accelerate AD progression and activate the defense cells to secrete pro-inflammation factors [17]. As is evident from the findings of this study, BSP effectively inhibited ROS production from the accumulated Aβ fibrils and downregulated the expression of inflammation-related genes in BV-2 cells, indicating the strong antioxidant and anti-inflammatory properties of BSP.

Oxidative stress and neuroinflammation are the two main factors involved in the progression of neurodegenerative diseases. Therefore, compounds with antioxidant and anti-inflammatory properties should provide significant neuroprotection. More and more evidence has shown that vitamins protect against central nervous system diseases in recent years. Like other neurodegenerative diseases, one of the leading causes of progressive neurodegenerative disorders in AD is oxidative stress [28]. There is increasing evidence that the antioxidant activity of vitamins may be beneficial in the treatment of AD. Therefore, vitamins have been used as adjuvants in the treatment of AD [29]. Vitamins are considered familiar and effective antioxidants. Studies have shown that a diet rich in antioxidant vitamins can slow the progression of Alzheimer’s disease. A meta-analysis indicated that vitamin A, B, C, D, and E deficiencies might increase the risk of AD [30]. Multiple pieces of evidence show that, compared with healthy individuals with complete neurocognitive functions, AD patients have reduced serum levels of VitA, VitB [31,32], VitC, VitD, VitE, and VitKin [33,34]. Interestingly, a cross-sectional and prospective study reported that the combination of VitC and VitE supplements reduced the prevalence and incidence of AD; however, they did not report VitA and found that VitB intake was not associated with AD [35].

Low levels of VitD in the serum are associated with increased risk of AD [36,37]. Calcium levels, parathyroid hormone, and specific cytokines regulate the concentration of the active form of VitD, calcitriol. The inactive form of VitD crosses the BBB, where it is converted into the active form by the enzyme CYP27B1 in glial and neuronal cells [38,39]. Microglia are also responsible for converting provitamin D into the active form of VitD [40]. Calcitriol controls the synthesis of nerve growth factors (NGF), and ultimately controls the differentiation and maturation of neuronal cells. In addition, calcitriol regulates the synthesis of glial cell line-derived neurotrophic factor (GDNF) [41,42]. Both NGF and GDNF regulate learning and memory through the hippocampal pathway. The concentration of amyloid precursor protein (APP) is effectively regulated by NGF [43]. Interruption of NGF signaling leads to an up-regulation of APP levels and an increase in aggregates in Abeta cells [44]. It is interesting that the active form of VitD and its analogs induce NGF expression [45], which suggests a possible mechanism by which VitD can indirectly improve the pathology of AD.

VitE plays an essential role in improving cognitive function and memory deficits [46]. Furthermore, VitE can be combined with other antioxidants to enhance the efficacy of treatment strategies effectively [47]. Although there is some evidence for the antioxidant activity of VitE, the role of VitE is controversial; clinical trials on VitE activity in AD have shown conflicting findings [48]. Research reported on the effect of VitE combined with donepezil supplementation on patients with mild cognitive impairment (MCI) (early AD) found that supplementation with VitE had no additional benefit [49]. MCI patients were treated with 2000 IU VitE daily and 10 mg donepezil or placebo daily for three years in this double-blind study. VitE does not affect MCI; however, donepezil reduced the rate of progression from MCI to AD in the first 12 months and reduced the longer duration of the apolipoprotein E4 carrier [50]. In short, since it is not clear whether long-term co-treatment with VitE is beneficial to the management of AD, a more comprehensive population study is needed.

Finally, since there are no international guidelines on dietary recommendations for AD, data on supplementation of vitamins A, B, C, D, and E are unclear [51]. Nevertheless, some clinical trials still support the potential role of vitamins in treating neurodegenerative diseases in the future. Stimulants are an essential factor and should be considered when designing vitamin dosages. Stimulants differentiate between beneficial and toxic activities of vitamins at specific doses. Compared with a single vitamin-based option, multivitamin supplements show therapeutic potential for neurodegenerative diseases. Multiple signaling pathways involve multiple vitamin methods that can enhance the antioxidant response [52].

Several studies have shown that aluminum exposure induces AD with strikingly similar clinical and pathological symptoms [53,54]. For instance, a study has shown that aluminum has been detected in both senile plaques and neurofibrillary tangle-bearing neurons in the brains of AD patients. In addition, neuropathological and neurobehavioral changes resulting in impaired learning ability induced by aluminum exposure are also evident from animal studies. Here, we demonstrated that AD rats lost cognition, with increased escape latency and reduction in the percentage of time spent in the target quadrant, which was recovered by treating the AD rats with the extracted BSP, suggesting the potential efficacy of BSP for ameliorating the working and learning memory impairment induced by AlCl_3_ in AD rats. The hippocampus is related to learning and memory; which is the initial site of Aβ accumulation [55].

BACE1 plays a key role in the production of Aβ; the digestion of APP by BACE1 is the first step in the chain reaction of Aβ aggregation, and its gene deletion has been shown to produce mild phenotypes. Several transcription-factor-binding sites are located within the BACE1 promoter region, including NF-κB, Sp1, and PPARγ, and the regulatory effects at the transcriptional level are probably involved in the expression of BACE1 [55]. Moreover, several groups have reported that BACE1 level and activity is increased in the AD brain [32,33], indicating that it presents an attractive therapeutic target for AD. Therefore, studies have attempted to identify factors that can suppress BACE1 expression [15]. In this study using an anti-BACE1 monoclonal antibody, we showed that BACE1 levels become elevated in the brains of AlCl_3_-induced AD rats, which was subsequently reduced in the BSP-treated rats, suggesting that BSP could be a potential source for the development of BACE1 inhibitor drugs.

Furthermore, western blot analysis of BACE1 and BACE1-staining supports the efficacy of BSP for alleviating the damages to the hippocampus and cerebral cortex in AlCl_3_-induced AD rats. The protective effects of BSP against AD could be attributed to its antioxidative and anti-inflammatory effects. The antioxidative effects of BSP are shown to be mediated through the nicotinamide adenine dinucleotide phosphate oxidase 4 (NOX4)/p22phox signaling pathway [21]. Therefore, it is speculated that BSP might be involved in the inhibition of the expression of NOX4 and p22phox, consequently blocking angiotensin II-induced ROS generation. However, further studies are required to delineate the exact underlying mechanism of action of BSP.

## 4. Materials and Methods

### 4.1. Materials

*B. striata* was obtained from Seng Chang Pharmaceutical Co., Ltd., (Taoyuan, Taiwan). Aβ42 peptides were synthesized by Gendanio Biotech Inc. (New Taipei City, Taiwan). 2,7-dichlorodihydrofluorescein diacetate (DCFDA) and aluminum chloride were purchased from Sigma Aldrich (St Louis, MO, USA). The RT-PCR primers were synthesized by MDBio Inc. (Taipei, Taiwan). The anti-BACE1 antibody and the Aβ (C-Terminal) antibody were purchased from Merck (Darmstadt, Germany) and Proteintech (Rosemont, IL, USA), respectively.

### 4.2. BSP Extraction and Purification

In the study, a new method was developed to extract BSP at low temperature by combining a cold extraction method with a vacuum system at 0.5 atm to retain the bioactivity of BSP and reduce protein contamination. Briefly, the as-received *B. striata* was chopped into pieces and ground to dry powder. Then 100 g *B. striata* dry powder was homogenized and dispersed by double-distilled water (1000 mL) in a low-pressure container at room temperature for 4 h [56]. The solution was then centrifuged at 5000 g for 10 min; the supernatant was collected and precipitated by adding 3000 mL 95% (*v*/*v*) ethanol and allowed to stand for overnight. Afterward, the solution was filtered using filter paper, and the precipitate was lyophilized and kept in a desiccator for later use. Subsequently, 100 g extracted dried powder was resuspended in 1800 mL distilled water and then mixed with 600 mL Sevage reagent (n-butanol: chloroform = 1:4); the solution was magnetically stirred overnight. On the next day, the mixture was centrifuged at 6000 rpm for 10 min, and the supernatant was collected and dialyzed using an MWCO-3500 dialyzer (60035515, Orange Scientific, Braine-I’ Alleud, Belgium) against double-distilled water to remove n-butanol. The final extract was lyophilized to obtain BSP.

### 4.3. The Characterization of Extracted BSP by Fourier-Transform Infrared (FTIR) Spectroscopy and Nuclear Magnetic Resonance (NMR)

The FTIR analysis was carried out by mixing 10 mg BSP with KBr in a ratio of 1:9 and then pressing it into a disc in an aluminum ring at 10 MPa. After that, the ring was mounted on an FTIR spectrophotometer (Spectrum 100 FTIR Spectrometer, PerkinElmer, Boston, MA, USA) at the wavenumber range of 450 to 4000 cm^−1^ and 400 nm/min scanning rate. The molecular structure of BSP was analyzed by ^1^H NMR and ^13^C NMR spectra measurements. BSP was dissolved by chloroform-d (CDCl_3_) at a concentration of 50 mg/mL, and then the spectra were recorded on a Bruker ARX-600 instrument (600 MHz, Bruker Co., Ltd., Fällanden, Switzerland) [56].

### 4.4. Preparation of Aβ Fibrils

Aβ42 peptides were purchased from Peptide Institute, Inc. (ASIA BIOSCIENCE CO., LTD., Taipei, Taiwan). The Aβ42 peptides were dissolved in hexafluoro-2-propanol (HFIP, Oakwood Products, Estill, SC, USA) for monomerization to obtain a final concentration of 1 mM. Then the Aβ42-HFIP solution was transferred to microfuge tubes in aliquots and kept at room temperature to evaporate HFIP, and then stored at −80 °C for later use. Immediately before use, the monomerized Aβ42 peptides in microfuge tubes were completely resuspended in 5 mM in anhydrous dimethyl sulfoxide (DMSO, catalog number D-2650, Sigma) by pipette mixing, and diluted to 100 μM with DMEM medium addition (Dulbecco’s modified Eagle’s medium, Sigma). The mixture was homogenized in a shaker at 37 °C for 7 days to aggregate into Aβ fibrils, the final concentration of Aβ fibrils was 100 μM [57,58].

### 4.5. Measurements of Thioflavin T Fluorescence

Thioflavin T (ThT), a commonly used probe to monitor Aβ fibril formation, emits fluorescence upon binding to Aβ fibrils and the intensity of fluorescence is used to measure the concentration of Aβ fibrils. To monitor the aggregation of Aβ42 into Aβ fibrils, 10 μM Aβ42 peptides were added to phosphate-buffered saline (PBS, pH 7.4) at 37 °C and transferred onto 96-well plates (Bio-One, Greiner, Kremsmünster, Austria) containing 10 μM ThT. The fluorescence intensity at 485 nm was measured by an ELISA reader (Infinite 200Pro, Tecan, Männedorf, Switzerland) under an excitation wavelength of 440 nm, after 1, 4, 7, 11, and 14 days [59].

### 4.6. Transmission Electron Microscopy (TEM)

400-mesh copper grid covered with Formvar and carbon (01754-F) was obtained from Ted Pella, INC, Taiwan. Ten microliters of Aβ42 (10 μM) were dropped onto the copper grids and dried at room temperature. It was then stained with 10 μL 1% (*w*/*v*) uranyl acetate (UA, 2%) for 20 s. The grid was washed with 10 μL distilled water twice and dried at room temperature. The images of aggregated Aβ fibrils at different time were examined using a transmission electron microscope (TEM, Hitachi H7650, Tokyo, Japan) operated at an acceleration voltage of 70 kV [60].

### 4.7. Evaluation of Cell Viability

The effects of BSP on cell viability were evaluated by the water-soluble tetrazolium (WST-1) assay (TaKaRa, Kusatsu, Shiga, Japan) using the N2a cell lines obtained from Bioresource Collection and Research Center (Hsinchu, Taiwan). The N2a cells were cultured in Dulbecco’s modified Eagle’s medium (DMEM, Sigma) containing 1% antibiotic-antimycotic (Gibco) and 10% fetal bovine serum (FBS, Hyclone, Logan, UT, USA) and incubated at 37 °C and 5% CO_2_ in a 95% humidified incubator. The overall process was based on the regulation of ISO 10993-5 [61]. Prior to the assessment, first, the extraction solution was obtained by soaking 1 mg/mL BSP in 10 mL culture medium for a day. In addition, the pre-cultured N2a cells were seeded onto 96-well plates with a density of 1 × 10^4^ cells/well and cultured for one day. Subsequently, the extraction solution was added to the culture wells and further cultured for one day. Following this, 200 μL WST-1 reagent was added to each well and incubated at 37 °C for 2 h. The OD value of each well was determined at 450 nm by using an ELISA plate reader (SpectraMax iD3, Molecular Devices, Inc, San Jose, CA, USA). The cell viability was calculated using the following formula:

(1) $$\mathrm{Cell}\;\mathrm{viability}\;\left(\%\right)=\frac{\left(\mathrm{OD}\;\mathrm{experiment}\;-\;\mathrm{OD}\;\mathrm{background}\right)\times 100}{\left(\mathrm{OD}\;\mathrm{control}\;-\;\mathrm{OD}\;\mathrm{background}\right)}$$

Zinc diethyldithiocarbamate (ZDEC, Sigma-Aldrich) and aluminum oxide (Sigma-Aldrich) were used as positive and negative controls, respectively. The cells cultured in only the fresh medium were used as the control group. The observations were repeated six times (*n* = 6) in each group.

### 4.8. Evaluation of Cytotoxicity

Cytotoxicity was evaluated using the LIVE/DEAD staining kit (L3224, Invitrogen, Waltham, MA, USA) according to the manufacturer’s instructions [62]. Briefly, 0.2 g specimen was immersed in 1 mL culture medium at 37 °C for 24 h, and the medium was used as the extracted medium for subsequent experiments. As described earlier, the pre-cultured N2a cells were seeded on a 24-well plate at a density of 2 × 10^4^ cells/well and incubated for 24 h. Afterwards, the extracted medium was added to each well, followed by the simultaneous addition of 10 μM Aβ fibrils and 1 mg/mL BSP, and cultured for 1 day. Subsequently, 2 μM calcein AM and 4 μM ethidium homodimer-1 (EthD-1) were added to each well and incubated at 37 °C for 30 min to stain the live and dead cells, respectively. Finally, the LIVE/DEAD staining was observed using a fluorescence microscope (IX81, Olympus, Tokyo, Japan) at excitation and emission wavelengths of 488 nm and 515 nm, respectively. N2a cells cultured in the extracted medium only were used as the control group. ZDEC and aluminum oxide were used as positive and negative controls, respectively.

### 4.9. Determination of Cellular ROS Generation

The ability of BSP to diffuse ROS induced by Aβ fibrils was measured by a DCFDA-cellular ROS detection assay kit (ab113851, Abcam, Cambridge, UK) [63]. In brief, N2a cells were seeded on 96-well plates at a density of 2.5 × 10^4^ cells/well and cultured for 1 day. Afterward, 1 mg/mL BSP and 10 μM Aβ fibrils were added to each well to induce ROS generation. On the next day, the medium was removed and washed with PBS. Subsequently, a culture medium containing 20 μM 2,7-dichlorodihydrofluorescein diacetate regent (DCFDA; Thermo Fisher, Waltham, MA, USA) was added to each well and further cultured for 45 min. The fluorescence intensity representing the ROS level induced by Aβ fibrils was detected using a multimode microplate reader (Molecular Devices, SpectraMax i3x, San Jose, CA, USA). The excitation and emission wavelengths were 495 and 535 nm, respectively.

### 4.10. Gene Expression Analysis

To assess the anti-inflammatory effects of BSP, we analyzed the expression levels of three inflammation-related genes— tumor necrosis factor-α (TNF-α), interleukin-6 (IL-6), and interleukin-10 (IL-10)—using BV-2 as target cells. The relative expressions of the genes were estimated from three experimental groups: (1) control group: BV-2 cells seeded on 96-well plate with a density of 1 × 10^5^ cells/mL were cultured without any treatment, (2) Aβ group: BV-2 cells at 1 × 10^5^ cells/mL were cultured and treated with 10 μM Aβ fibrils, and (3) Aβ-BSP group: BV-2 cells at 1 × 10^5^ cells/mL were cultured and treated with 1 mg/mL BSP and 10 μM Aβ fibrils. Total RNA was extracted using Qiazol reagent (Qiagen, Valencia, CA, USA) following the manufacturer’s protocol. Random hexamers (Vivantis Inc., California, CA, USA) and reverse transcriptase (Vivantis Cat No: RTPL12) were used for the first-strand cDNA synthesis with the following PCR parameters: 95 °C for 3 min (denaturation), 40 cycles of 95 °C for 20 s, 60 °C for 30 s (annealing), and 72 °C for 30 s (elongation). The real-time RT-PCR was performed using the TOOLS 2X SYBR qPCR Mix (Biotools Co., Ltd., Taipei, Taiwan) on a CFX Connect Real-Time PCR Detection System (Bio-Rad, Hercules, CA, USA). The specific primers (Biotools Co., Ltd., Taipei, Taiwan) used for real-time RT-PCR are shown in Table 1. The changes in expression of the target genes were calculated using GAPDH as an endogenous control [64].

### 4.11. AlCl^3-^Induced AD Rat Model to Develop Alzheimer’s Disease (AD)

Eight-week-old Sprague Dawley (SD) male rats were purchased from BioLASCO Taiwan Co., Ltd. (Taipei City, Taiwan) and adapted to the new environment for two weeks. All experiments were carried out in compliance with the National Taiwan University College of Medicine’s Institutional Animal Care and Use Committee (IACUC no. 20130429). We maintained the animals according to the Guide for the Care and Use of Laboratory Animals. The behavioral tests performed were approved by the Animal Ethics Committee of the National Taiwan University Hospital, Taiwan. AD was induced by intraperitoneal (IP) injection of AlCl_3_ (100 mg/mL AlCl_3_ dissolved in normal saline) three times a week as described in a previous study [65]. Animal models play a central role in AD research [66]. Long-term exposure to aluminum (Al^3+^) is considered a pathogenic factor in the pathogenesis of AD [67]. Previous animal studies have shown that Al^3+^ induces neuropathological, neurochemical, and neurobehavioral changes similar to AD [22]. In addition, it has been reported that Al^3+^ is found in SP and neurons with neurofibrillary tangles in the brains of AD patients [67]. Therefore, Al^3+^ administration can be used to induce AD in animal models. The SD rats (*n* = 18) were randomly categorized into 3 groups with 6 rats per group: (1) Control group: rats injected with normal saline, (2) AlCl_3_ group: rats injected with AlCl_3_ (100 mg/kg body weight) three times every week and consecutively for eight weeks, (3) AlCl_3_-BSP group: rats were injected with AlCl_3_ (100 mg/kg body weight), and then treated with BSP (10 mg/kg/day) by oral administration during the AD induction period. At the end of the experiment (18-week-old SD rats after purchasing), the rats were subjected to the Morris Water Maze test to assess their memory and cognitive functions. The blood was collected to assess the blood elements, and serological analysis to check the safety. The cortex and hippocampus were harvested after sacrificing the rats by euthanasia for western blotting and histological examination.

### 4.12. Morris Water Maze Test

The Morris Water Maze was used to assess working and spatial memory retention in the rats, following a previous study [68]. Briefly, the circular pool (160 cm in diameter and 35 cm in height) was divided into four quadrants. It contains a 10 cm (diameter) × 25 cm (height) round escape platform, the same color as the rest of the basin (to eliminate any false-positive results due to vision), placed in the constant quadrant of the basin during the entire test kept 2 cm below the water surface. Animals had 4 trials per day separated by 10 min for 4 successive days. For each training session, the rat was gently placed in water at a different drop location and allowed to find the submerged platform; the rat was guided toward the platform if it could not find the platform within 2 min. Once the platform was reached, the rat was allowed to stay on it for 30 s. The training was continued in each quadrant for four consecutive days. After four training sessions, the time taken by the rat to reach the escape platform was recorded using the EthoVision software (The Observer XT, Noldus Information Technology, Wageningen, Netherlands). The retrieval tests of working and spatial memories were performed as described in a previous study [69]. To assess spatial memory, we recorded the time taken (s) by each rat to reach the escape platform from their initial position, whereas working memory was assessed from the time spent (s) by the rat in the same quadrant (maximum 120 s) without the escape platform [70].

### 4.13. Blood Analysis

The safety in vivo was evaluated by blood element analysis and serological analysis. Blood from the rats was collected by cardiac punctures at the end of the experiment. Serum was obtained by centrifuging the collected blood samples at 1500 rpm at 4 °C for 15 min. The collected serum samples were stored at −80 °C for subsequent analyses. For biochemical tests, alanine aminotransferase (ALT), aspartate aminotransferase (AST), blood urea nitrogen (BUN), and lactate dehydrogenase (LDH) in serum were measured following a previously described method [36]. For blood element analysis, red blood cells (RBC), hemoglobin (HGB), hematocrit (HCT), mean cell volume (MCV), mean corpuscular hemoglobin (MCH), mean corpuscular hemoglobin concentration (MCHC), reticulocytes (RET), platelets (PLT), white blood cells (WBC), neutrophil (NEUT), lymphocytes (LYMPH), monocytes (MONO), eosinophils (EO), and basophils (BASO) were measured. Blood element and serum were measured by the National Taiwan University Veterinary Hospital, Taiwan. Reference: Charles River Laboratories, CD^®^ IGS Rat Model Information Sheet [71,72].

### 4.14. Western Blotting

The total protein was extracted from the hippocampus and cortex tissues of the experimental animals using RIPA lysis buffer (Thermo Fisher, Waltham, MA, USA) containing a protease inhibitor cocktail. The protein concentration was measured by the Bradford protein assay kit (Z5030028, BioChain Institute Inc., San Francisco CA USA). The samples were resolved with equal amounts of protein (25 µg) using 12% SDS-PAGE. Three samples from each group were washed, lysed, and equal amounts of protein were separated and transferred onto a polyvinylidene difluoride membrane (Millipore, Burlington, MA, USA), blocked, and incubated with primary antibodies against BACE1 and β-actin (Anti-BACE1, cat. no. ab183612; dilution, 1:500, and anti-β-actin, T5168, Sigma-Aldrich; dilution, 1:5000). BACE1 protein was visualized by enhanced chemiluminescence [73].

### 4.15. Histological Analysis and Immunohistochemical (IHC) Staining

The cortex and hippocampus harvested from the experimental rats were treated with a series of alcohol and fixed by 4% glutaraldehyde. The specimens were then bisected and embedded in paraffin. Subsequently, the paraffin-embedded tissue blocks were cut into 5-μm-thick sections, placed on the glass slides, and stained with BACE1 immuno-histochemical anti-body. Hematoxylin and eosin (H&E) stain was used for contrast imaging. The slides were then deparaffinized and rehydrated with 0.1% hydrogen peroxide (Sigma-Aldrich, St. Louis, MO, USA) in PBS solution for 10 min to block endogenous peroxidases. For retrieval, nonspecific background staining was blocked by 20 μg/mL proteinase K (Sigma-Aldrich, USA) solution [74]. The solution containing the slides was incubated in a humidified chamber at 37 ºC for 20 min. The primary antibodies against BACE1 (Anti-BACE1, cat. no. ab183612), were diluted with 1% BSA in a ratio of 1:500 (*v*/*v*). The diluted primary antibody was then applied to the slides and incubated at 4 °C overnight. After incubation, the tissue sections were washed by TBS containing 0.025% Triton-X 100 with gentle agitation. The sections were further incubated in 1% BSA containing goat anti-rabbit HRP IgG secondary antibody at 1:5000 (*v*/*v*) dilution. Finally, the sections were further treated with 3, 3′-diaminobenzidine (DAB, Sigma-Aldrich, USA) substrate solution as an enhancer to more clearly reveal colors under the optical microscope [75].

### 4.16. Statistical Analyses

The data were expressed as mean ± standard deviation (SD). All statistical analyses were done using Student’s *t*-test or one-way analysis of variance (ANOVA) post hoc tests and by Tukey’s test using GraphPad Prism software (Prism 9, GraphPad Software, Inc., La Jolla, CA, USA), where *p*-values of less than 0.05 were considered statistically significant [76].

## 5. Conclusions

In this study, BSP was successfully isolated and purified from dry *B. striata* by a modified extraction method. The molecular structure and characterized functional groups, identified and confirmed by NMR and FTIR, respectively, are consistent with those reported in previous studies. Though previous studies have shown its antioxidant and anti-inflammatory effects, this study, for the first time, investigated the role of BSP in AD therapy. The findings demonstrated that the extracted BSP could effectively diffuse ROS from the Aβ fibrils and had good biocompatibility with N2a cell lines, whereas its anti-inflammatory ability was evident in BV-2 cell lines. It also suppressed the expression of BACE1, a prime therapeutic target for the development of drugs to treat AD. Moreover, BSP treatment in AD rats reduced the damage to the hippocampus and cortex and recovered the neurodegeneration induced by AlCl_3_. Collectively, the findings of this study indicate that BSP could be a potential therapeutic agent in AD treatment.

## Figures and Tables

**Table 1:** Primers used for gene expression analysis.

Name	Sequence
TNF-α-Forward	5′-CATCTTCTCAAAATTCGAGTACAA-3′
TNF-α-Reverse	5′-TGGGAGTAGACAAGGTACAACCC-3′
IL-6-Forward	5′-GGAGCCCACCAAGAACGATAGTCA-3′
IL-6-Reverse	5′-GAAGTAGGGAAGGCCGTGGTT-3′
IL-10-Forward	5′-TAAGGCTGGCCACACTTGAG-3′
IL-10-Reverse	5′-GTTTTCAGGGATGAAGCGGC-3′
GAPDH-Forward	5′-TGCTGAGTATGTCGTGGAGTCT-3′
GAPDH-Reverse	5′-AATGGGAGTTGCTGTTGAAGTC-3′

## Data Availability

The datasets used and/or analyzed in the current study are available from the corresponding author on reasonable request.
